# Bovine pestivirus is a new alternative virus for multiple myeloma oncolytic virotherapy

**DOI:** 10.1186/s13045-020-00919-w

**Published:** 2020-07-11

**Authors:** Valentina Marchica, Valentina Franceschi, Rosanna Vescovini, Paola Storti, Emanuela Vicario, Denise Toscani, Alessia Zorzoli, Irma Airoldi, Benedetta Dalla Palma, Nicoletta Campanini, Eugenia Martella, Cristina Mancini, Federica Costa, Gaetano Donofrio, Nicola Giuliani

**Affiliations:** 1 Department of Medicine and Surgery, University of Parma, Parma, Italy; 2 Department of Medical-Veterinary Science, University of Parma, Parma, Italy; 3 Stem Cell Laboratory and Cell Therapy Center, IRCCS “Istituto Giannina Gaslini”, Genoa, Italy; 4 Hematology, “Azienda Ospedaliero-Universitaria di Parma”, Parma, Italy; 5 Pathology, “Azienda Ospedaliero-Universitaria di Parma”, Parma, Italy

**Keywords:** Multiple myeloma, Oncolytic virus, Bovine viral diarrhea virus, Oncolytic virotherapy

## Abstract

**Background:**

The oncolytic viruses have shown promising results for the treatment of multiple myeloma. However, the use of human viruses is limited by the patients’ antiviral immune response. In this study, we investigated an alternative oncolytic strategy using non-human pathogen viruses as the bovine viral diarrhea virus (BVDV) that were able to interact with CD46.

**Methods:**

We treated several human myeloma cell lines and non-myeloma cell lines with BVDV to evaluate the expression of CD46 and to study the effect on cell viability by flow cytometry. The possible synergistic effect of bortezomib in combination with BVDV was also tested. Moreover, we infected the bone marrow mononuclear cells obtained from myeloma patients and we checked the BVDV effect on different cell populations, defined by CD138, CD14, CD3, CD19, and CD56 expression evaluated by flow cytometry. Finally, the in vivo BVDV effect was tested in NOD-SCID mice injected subcutaneously with myeloma cell lines.

**Results:**

Human myeloma cells were selectively sensitive to BVDV treatment with an increase of cell death and, consequently, of apoptotic markers. Consistently, bone marrow mononuclear cells isolated from myeloma patients treated with BVDV, showed a significant selective decrease of the percentage of viable CD138^+^ cells. Interestingly, bortezomib pre-treatment significantly increased the cytotoxic effect of BVDV in myeloma cell lines with a synergistic effect. Finally, the in vitro data were confirmed in an in vivo myeloma mouse model showing that BVDV treatment significantly reduced the tumoral burden compared to the vehicle.

**Conclusions:**

Overall, our data indicate, for the first time, a direct oncolytic effect of the BVDV in human myeloma cells suggesting its possible use as novel alternative anti-myeloma virotherapy strategy.

## Background

Multiple myeloma (MM) is a hematological malignancy characterized by the accumulation of plasma cells (PCs) in the bone marrow (BM) microenvironment that critically supports MM cell growth and survival [1]. Despite the significant therapeutic progress due to the introduction of several new drugs, MM remains an incurable disease [2, 3].

The oncolytic virotherapy is an alternative therapeutic strategy in cancer treatment, exploiting natural or genetically engineered viruses able to infect, transduce, and consequently kill cancer cells directly or indirectly through the delivery by the microenvironment cells [4, 5]. Moreover, it has been shown that the oncolytic virus may increase the sensitivity of tumor cells to immunotherapy [6–8].

Several human oncolytic viruses, such as measles virus (MV), vesicular stomatitis virus, reovirus, and adenovirus have shown promising results for the treatment of MM and are currently considered as potential cancer therapeutics [9]. These oncolytic viruses have been investigated pre-clinically as monotherapy, as combination therapy in conjunction with chemotherapy and/or radiation therapy, and as purging agents during autologous stem cells transplantation [9]. In particular, MV is the most comprehensively studied oncolytic virus for MM and the first virus underwent to phase I clinical trial investigation for this disease [10]. Other naturally occurring viruses, such as adenovirus that is currently undergoing phase III clinical trial for solid tumors, are anticipated to undergo a phase I clinical trial for MM in the near future [11–13]. Overall, these data suggest that the oncolytic virotherapy could be a promising novel alternative anti-MM strategy.

However, the use of human viruses is limited by the antiviral immune response of the patients due to vaccination or natural infection, as suggested also by preliminary data on MM patients treated with MV [14]. To enhance the therapeutic efficacy of virotherapy, in this project, for the first time we investigated the use of a bovine viruses as alternative oncolytic strategy in MM. In particular, we show the anti-MM activity of bovine viral diarrhea virus (BVDV), known to bind CD46 receptor, as reported for human MV [15, 16]. BVDV is a single-stranded RNA virus, belonging to Pestivirus genus and Flaviviridae family, considered one of the major viral pathogens of cattle, directly associated with mucosal disease [17, 18]. It is known that BVDV, in bovine models, induces cell death by apoptosis due to an increase of intracellular viral RNA accumulation [19, 20], but its oncolytic activity has never been reported in human cancers.

## Methods

### Cells lines and reagents

#### Cell lines

The human myeloma cell lines (HMCLs) JJN3, OPM2, INA-6, MM1.S, NCI-H929, the T-acute lymphoblastic leukemia cell line (T-ALL) SKW3-KE37 and the B-acute lymphoblastic leukemia cell lines (B-ALL) NALM-6, and the lymphoma cell lines GRANTA-519 and RAJI were purchased from Leibniz Institute Deutsche Sammlung von Mikroorganismen und Zellkulturen GmbH (Braunschweig, Germany). The B-ALL cells RS4;11, the T-ALL cells SUP-T1 were purchased from ATCC (Manassas, VA, USA). Cells were maintained in RPMI-1640 medium supplemented with 10% fetal bovine serum (FBS), l-glutamine (2 mM), amphotericin B (0.25 μg/mL), and antibiotics (100 U/mL penicillin, and 100 μg/mL streptomycin) (ThermoFisher Scientific, Monza, Italy).

#### Bovine viruses

Bovine herpesvirus-4 type 4 (BoHV-4-A-EGFPΔTK) [21] and bovine viral diarrhea virus (BVDV, strain NADL, ATCC) were propagated by infecting confluent monolayers of bovine embryo kidney [(BS CL-94) BEK] or Madin Darby Bovine Kidney cells [(ATCC: CCL-22) MDBK] at a multiplicity of infection (MOI) of 0.5 50% tissue culture infectious doses (TCID_50_) per cell and maintained in MEM (ThermoFisher Scientific) with 2% FBS (ThermoFisher Scientific) for 2 h. The medium was then removed and replaced by fresh MEM containing 10% FBS. When approximately 90% of the cell monolayer exhibited cytopathic effect (CPE) (approximately 72 h post-infection), the virus was prepared by freezing and thawing cells three times and pelleting the virions through 30% sucrose, as described previously [22]. Virus pellets were resuspended in cold MEM without FBS. TCID_50_ were determined in BEK or MDBK cells by limiting dilution.

#### Drug

Bortezomib (Bor) was purchased from Selleckchem (Munich, Germany). The drug was reconstituted following the manufacturer’s protocol and diluted in the cell culture medium just before the use*.*

### Patient’s samples

A total cohort of 31 consecutive patients (13 males and 18 females) with malignant PC disorders were included in the study: 2 plasma cell leukemia (PCL) (median age 63 years, range 53–73), 29 with active MM including 18 newly diagnosed MM (ND-MM) (median age 74 years; range 52–86) and 11 relapsed MM (R-MM) (median age 73 years; range 59–81). All patients were diagnosed according to the International Myeloma Working Group (IMWG) revised criteria [23]. The main clinical characteristics of all the patients enrolled in the study are summarized in Table 1.

**Table 1:** Clinical characteristc of patients

Diagnosis	Stage	ISS	Gender	Age	Light chains	%PC BOM	High risk
MM-1	ND	III	M	76	l	90%	No
MM-2	ND	II	F	73	l	60%	No
MM-3	ND	II	F	52	k	70%	No
MM-4	ND	II	M	85	k	30%	Yes
MM-5	ND	III	M	80	k	70%	No
MM-6	ND	III	F	72	l	70%	Yes
MM-7	ND	III	F	74	k	18%	No
MM-8	ND	II	M	74	k	40%	No
MM-9	ND	III	M	57	l	80%	
MM-10	ND	III	F	71	k	100%	Yes
MM-11	ND	I	F	80	k	30%	No
MM-12	ND	III	M	79	k	25%	No
MM-13	ND	II	F	67	k	60%	No
MM-14	ND	II	M	77	l	30%	
MM-15	ND	III	F	86	l	80%	No
MM-16	ND		F	57	k	25%	No
MM-17	ND	III	F	74	k	70%	Yes
MM-18	ND	I	F	53	k	30%	Yes
MM-19	R	III	F	81	k	40%	Yes
MM-20	R	III	M	78	l	40%	Yes
MM-21	R	I	M	59	k	20%	
MM-22	R	I	M	65	k	60%	No
MM-23	R	III	M	78	k	85%	Yes
MM-24	R	–	F	79	l	30%	No
MM-25	R	III	F	72	l	90%	
MM-26	R	III	F	79	k	80%	Yes
MM-27	R	I	M	69	l	50%	No
MM-28	R	I	F	72	k	50%	No
MM-29	R	III	M	73	k	25%	Yes
PCL-1	D	III	F	53	k	90%	No
PCL-2	R	III	F	73	l	90%	Yes

Abbreviations: *MM* multiple myeloma, *ND* newly diagnosed, *R* relapsed, *F* female, *M* male, *ISS* International Staging System, *%PC BOM* percentage of plasma cells evaluated by bone biopsy, *high risk* defined by presence of deletion of 17P and or traslocation (t) of (4;14) and or t(14;16)

BM aspirates were obtained from the iliac crest of patients after informed consent according to the Declaration of Helsinki. Total BM mononuclear cells (MNCs) were obtained from BM aspirates by Ficoll-Hypaque (Bichrome AG, Berlin, Germany) density sedimentation and cultured in RPMI 1640 medium supplemented with 20% FBS, in penicillin (100 U/ml), streptomycin (100 μg/ml), l-glutamine (2 mM), and fungizone antimycotic (2.5 μg/ml); all purchased from ThermoFisher Scientific. This study was approved by local ethic committee institutional review board of Parma (Parma, Italy).

### Viruses and drug treatments

The HMCLs, T-ALL, and B-ALL cell lines were treated with BVDV or vehicle or heat-inactivated BVDV and maintained at 37 °C in a 5% CO_2_ atmosphere, for 24, 48, and 72 h. Heat inactivated BVDV is obtained after 1 h treatment at 95 °C. For in vitro experiments, we used 1 MOI of BVDV/1 × 10^6^ cells. The same experiments were performed with or without 0.05% trypsin-EDTA (ThermoFisher Scientific) incubation and after treatments all cells were collected for Multiplex PCR analysis. In addition, JJN3, OPM-2, and INA-6 were also treated with BoHV-4 or vehicle or heat-inactivated BoHV-4 for the same time course and at the same MOI.

The HMCL JJN3 cells were also pre-treated with Bor (2.5 nM) or vehicle for 24 h. Following drug washout with PBS, cells were counted and infected with BVDV for 24, 48, and 72 h. At the end of experiments, cells were collected for flow cytometry analysis. For combination index experiments, JJN3 cells were pre-treated with Bor at different concentrations (0.125–8 nM) for 24 h, washed out with PBS and incubated in 96-well plates with or BVDV at several viral titers (0.0625–4 MOI) or the combination of the 2 drugs (2:1) or vehicle for 48 h. MTT assay was assessed to calculate the effect of combination of the 2 drugs. The combination index analysis was performed using CompuSyn software version 1 (http://combosyn.com/).

BM MNCs from patients were cultured with or without BVDV for 72 h and maintained in at 37 °C in a 5% CO_2_ atmosphere. After treatment, all cells were collected for flow cytometry analysis, PCR analysis, and western bot analysis.

### Flow cytometry

#### CD46 expression

Expression levels of CD46 antigen were determined on HMCLs, B and T-ALL, lymphoma cells, and on BM MNCs obtained from MM patients by flow cytometry analysis and expressed as median fluorescence intensity (MFI). In particular, to evaluate the expression of CD46, 0.2 × 10^6^ HMCLs or non-MM cells were stained with a saturating quantity of anti-CD46 PerCP (Thermofisher Scientific) for 30 min at 4 °C protect from light. Cells were then washed with a cell wash solution (PBS plus 5% human serum albumin and 5 w/V sodium azide) and directly analyzed by flow cytometry.

CD46 expression levels on fresh BM MNCs were detected by staining 0.5 × 10^6^ cells/tube with saturating quantities of antibodies (all, except anti-CD46, purchased from BD Bioscience, Franklin Lakes, NJ, USA) combined in the following two panels: (1) anti-CD56 FITC, anti CD138 PE, anti-CD46 PerCP, and anti-CD3 APC; (2) anti-CD14 FITC, anti-CD138 PE, anti-CD46 PerCP, and anti-CD19 APC. After incubation for 30 min, at 4 °C protection from light, BM MNCs were washed with the cell wash solution and analyzed by flow cytometry. Unstained samples were employed for gating controls. Concerning flow cytometry gating strategy, the analysis included a forward (FSC) and side (SSC) scatter gating to identify the cells of interest based on the relative size and complexity of the cells, while removing debris and cell fragments. In BM MNCs analysis, CD46 expression levels were determined on specific gates identifying: T lymphocytes (CD3+), B lymphocytes (CD19+), monocytes (CD14+), NK cells (CD56+CD138−), and MM cells (CD138+).

#### Viability staining and apoptotic assay on cell lines

HMCLs, T and B ALL cells, and lymphoma cells were stained, according to manufacturer’s instructions, with 7-Amino Actinomycin D (7-AAD) purchased from BD Biosciences (Franklin Lakes, NJ, USA). Viable and non-viable cells were identified as 7-AAD-negative or 7-AAD-positive events, respectively, in dot plots of SSC vs. 7-AAD.

Apoptosis was assessed by the APO2.7 assay, which specifically detects 7A6, a 38-kDa mitochondrial membrane antigen expressed during apoptosis. After treatment, cells were collected, stained with saturating quantity of PE-conjugated APO2.7 antibody (Beckman Coulter, Marseille, France), and analyzed by flow-cytometry.

#### Identification of BM MNCs subsets

After treatments, BM MNCs were collected and stained with saturating quantities of antibodies (purchased from BD Bioscience) combined in the following two panels: (1) anti-CD14-FITC, anti-CD138-PE, and anti-CD19-APC; (2) anti-CD56-FITC, anti-CD138-PE, and anti-CD3-APC. Before the acquisition, 7-AAD was added to staining panels according to manufacturer instructions. The gating strategy to evaluate the percentage of viable MM cells (CD138^+^), T lymphocytes (CD3^+^), B lymphocytes (CD19^+^), monocytes (CD14^+^), and NK cells (CD56^+^CD138^-^) included a first FSC and SSC gating to identify the cells of interest. In particular, we analyzed MM cells (CD138+), T lymphocytes (CD3+), B lymphocytes (CD19+), monocytes (CD14+), and NK cells (CD56+CD138−) based on the relative size and complexity of the cells, while removing debris and cell fragments, and a subsetting live gating based on 7-AAD negative expression.

The BVDV oncolytic effect on MM cells was calculated using the following formula: % of CD138^+^ cells mortality = 1−(% of CD138^+^ 7-ADD^−^ in BVDV condition/CD138^+^ 7-AAD^−^ in control condition) × 100. In all flow cytometry procedures, the acquisition and analysis of samples were performed on a two-laser FACSCalibur instrument (BD Biosciences) using CellQuest software (BD Biosciences).

### Reverse transcriptase PCR amplification and nested multiplex PCR

#### RNA isolation

Total cellular RNA was extracted from cells using RNeasy total RNA isolation kit (Qiagen; Hilden, Germany) following the manufacturer’s instructions, and then quantified using a NanoDrop™ One (ThermoFisher Scientific).

For the RNA viral gene NS5B detection, reverse transcription (RT) and PCR were combined in a single step as previously described [24].

Primary PCR was performed using the following specific primer pairs:

SENSE A: 5′-AAGATCCACCCTTATGA(A/G)GC-3′

ANTISENSE A: 5′-AAGAAGCCATCATC(A/C)CCACA-3′

The product of the primary PCR was used in nested PCR. The multiplex primers used for nested PCR are the following:

BVDV-1: 5′-TGGAGATCTTTCACACAATAGC-3′

MULTISENSE: 5′-GCTGTTTCACCCAGTT(A/G)TACAT-3′

For internal sample quality control, a volume of 1 μg of RNA was reverse-transcribed, in accordance with the manufacturer’s protocol. Qualitative PCR were performed using the following specific primer pairs for GAPDH:

F: 5′-CAACGGATTTGGTCGTATTG-3′

R: 5′-GGAAGATGGTGATGGGATTT-3′

Products were electrophoresed on a 1.5% agarose gel (ThermoFisher Scientific) and stained with gel red (Biotium, Hayward, USA).

### Western blot

The cytosolic extracts were obtained using a commercial kit (Active Motif, Carlsbad, CA, USA) following the manufacturer’s protocol. For immunoblotting, the following antibodies were used: mouse monoclonal anti-caspase 3 antibody (Active Motif, Carlsbad, CA, USA), rabbit monoclonal anti-Mcl-1 antibody (Cell Signaling, Leiden, Netherlands), rabbit monoclonal anti-Bcl-2 antibody (Cell Signaling, Leiden, Netherlands), and mouse monoclonal anti-β-actin (Sigma-Aldrich, Milan, Italy) as internal control. The secondary antibodies peroxidase conjugated were anti-mouse (BD Pharmingen, Franklin Lakes, NJ, USA) and anti-rabbit (Cell Signaling). Protein bands were quantified using ImageJ software (U.S. National Institutes of Health, Bethesda, MA, USA).

### In vivo mouse studies

Two different groups of six severe combined immunodeficiency/non-obese diabetic (NOD/SCID) mice (4 to 6 weeks old) were housed under specific pathogen-free conditions and were injected subcutaneously with 5 × 10^6^ of JJN3. When plasmacytomas have become palpable, BVDV or saline solution was injected intratumorally twice a week for 2 weeks. All procedures were performed according to the National and International current regulations. Tumor growth was monitored at different time points and, 3 weeks after cell inoculation, mice were killed and tumor mass, spleens, and peripheral blood were collected for immunohistochemical staining and western blot analysis. Maximum length, thickness, and width of the tumor masses were measured with a caliper, and tumor volume was calculated according to the following formula: 0.523 × length × width^2^. Plasmacytomas obtained from tumors removed from mice were fixed in 10% neutral buffered formalin, embedded in paraffin, and stained with hematoxylin and eosin. Moreover, plasmacytomas lysates were used to perform the western blot analysis. This study was approved by the Italian Ministry of Health review board (Italy).

### Statistical analysis

Data were expressed as mean ± SD. ANOVA and two-tail Student’s *t* tests or Kruskal-Wallis and Mann-Whitney tests were used, and *p* values < 0.05 were considered statistically significant. GraphPad Prism 8™ (GraphPad Software Inc., La Jolla, CA, USA) was used for all the statistical analyses.

## Results

### BVDV treatment selectively leads to HMCLs death

**Fig. 1:**
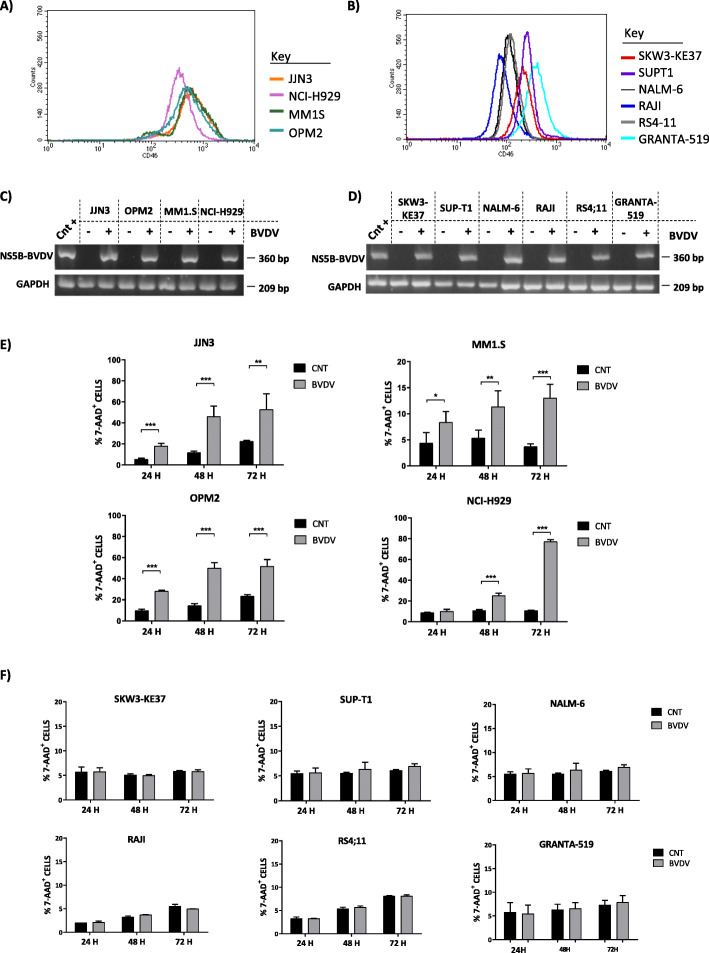
Expression levels of CD46 and oncolytic effect of BVDV on several hemopoietic cancer cell lines. Representative histogram plots of flow cytometry showed CD46 expression levels on **a** four HMCLs (JJN3, NCI-H929, MM.1S, and OPM2) and **b** two T-ALL lines as SKW3-KE37, SUP-T1, two B-ALL lines as NALM-6, RS4;11, and two B cell lymphomas lines as GRANTA-519, RAJI. The graphs represent the CD46 median fluorescence intensity (MFI). The picture shows the presence of BVDV in MM cell lines (c) and in non-MM cell lines (d) evaluated by Nested multiplex PCR after 24 h of BVDV (1 MOI) treatment. GAPDH was used as internal quality control. e The histograms represent the percentage of 7-AAD^+^cells after 24, 48, and 72 h of treatment with BVDV (1 MOI). We reported the mean ± SD percentage of dead cells, as 7-AAD^+^ cells, of four independent experiments on JJN3, MM1.S, OPM2, and NCI-H929 and (f) three independent experiments of non-MM cells (SKW3-KE37, SUP-T1, NALM-6, RS4;11, GRANTA-519, RAJI); *p* values were calculated by two-tailed Student’s *t* test. (*p < 0.05, **p < 0.01, ***p < 0.001) (CNT = control, untreated cells)

Firstly, we analyzed the expression levels of CD46, the cellular receptor for BVDV entry, on HMCLs, and B-ALL, T-ALL, lymphoma cell lines (defined as non-MM cells) by flow cytometry. In line with literature data, all cell lines were CD46-positive [25]. Interestingly, we observed that MM cells express higher levels of CD46 (Fig. 1a) (median MFI_CD46_ value 523.74) than non-MM cells (median MFI_CD46_ value 161.61) (Fig. 1b), suggesting that MM cells could be more susceptible to BVDV effect.

To verify our hypothesis, the BVDV oncolytic effect was assessed on the same MM and non-MM cell lines after 24, 48, and 72 h of treatment. The infection efficiency, in terms of viral gene expression, was checked after 24 h by nested multiplex PCR. As reported in Fig. 1c and d, the presence of BVDV was observed both in MM cells and non-MM cells, respectively.

Subsequently, flow cytometry analysis on HMCLs treated with BVDV reported a significant increase of cell mortality, as percentage of 7-AAD^+^ cells, already after 24 h of infection for JJN3, OPM2, and MM1.S, and after 48 h for NCI-H929 (Fig. 1e). In Table 2, we reported the mean ± SD% of dead cells of HMCLs treated with BVDV vs. untreated cells. Moreover, as expected, the heat-inactivated BVDV does not affect cell viability (Supplemental Figure S1A), comparable to the untreated cells (control in all experiments), and we have not observed the presence of viral genes. (Supplemental Figure S1B).

**Table 2:** Statistical analysis on HMCLs treated with BVDV

		Mean ± SD % of dead cells BVDV vs. CNT	*p* value
JJN3	24 h	18.5 ± 2.5% vs. 5.3 ± 1%	0.00005
	48 h	44.2 ± 9.8% vs. 11.3 ± 1.5%	0.0002
	72 h	54.3 ± 12.8% vs. 21.5 ± 1.9%	0.002
OPM2	24 h	28 ± 1% vs. 9 ± 1.4%	< 0.00001
	48 h	48 ± 5.6% vs. 13 ± 2.7%	0.00002
	72 h	58 ± 1.7% vs. 22 ± 1.8%	< 0.00001
MM1.S	24 h	9 ± 1.8% vs. 4 ± 1.7%	0.012
	48 h	12 ± %3 vs. 5 ± 1.2%	0.005
	72 h	20 ± 1.7% vs. 4 ± 0.5%	< 0.00001
NCI-H929	24 h	10 ± 0.01 vs. 8 ± 0.01	0.16553
	48 h	26 ± 2.6% vs. 10 ± 1.2%	0.00004
	72 h	76 ± 2.5% vs. 10.5 ± 0.5%	< 0.00001

Abbreviations: *BVDV* bovine viral diarrhea virus, *CNT* control (untreated cells), *SD* standard deviation

The increase of cell mortality after BVDV treatment was not observed in non-MM cells, denoting that the lytic effect of BVDV is specific for MM cells (Fig. 1f). To demonstrate the critical role of the CD46 receptor of BVDV, we used an oncolytic virus known to lack to interaction with CD46. Interestingly, we did not find any significant cytotoxic effect of BoHV-4 treatment in all the HMCLs tested at the different time course. Supplemental Figure S2 reported one representative experiment of JJN3 treated with BoHV-4 for 24, 48, and 72 h, evaluated by flow cytometry.

Moreover, to better investigate the mechanism of BVDV lytic effect, we treated JJN3, SUPT-1, GRANTA-519, and NALM-6 cell lines with 1 MOI of BVDV for 48 h. At the end of the culture period, in order to remove the virus attached to the cellular surface, cells were collected with or without trypsin incubation. Focusing on BVDV treated cells, we found that BVDV viral gene expression was not detectable in non-MM cell lines after trypsin incubation (Supplemental Figure S3). On the other hand, we observed the expression of BVDV viral gene in MM cells with and without trypsin incubation. These results suggest that BVDV binds to both MM and non-MM cells but is able to entry only in MM cells.

### BVDV triggers apoptosis in HMCLs

**Fig. 2:**
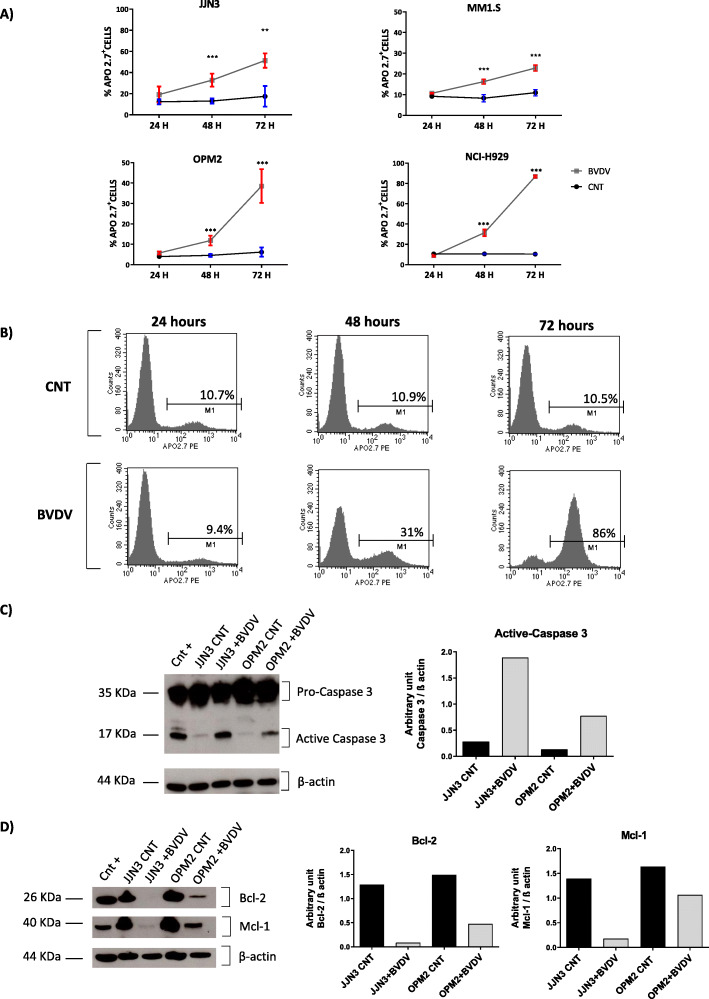
The cytotoxic *in vitro* effect of BVDV on HMCLs. **a** Mean ± SD of the percentage of Apo 2.7^+^ cells in JJN3, MM1.S, OPM2, and NCI-H929 after 24, 48, and 72 h of treatment with BVDV (1 MOI). The graphs represent the mean percentage of Apo2.7^+^ cells of four independent experiments for each cell line evaluated by flow cytometry. **b** Representative histogram plots of flow cytometry showing the percentages of NCI-H929 cells positive for the apoptotic marker APO2.7, after 24, 48, and 72 h of BVDV (1 MOI) treatment or in the control condition. **c** Pro- and active-caspase 3 expression was evaluated by western blot in JJN3 and OPM2 cells treated with or without BVDV (1 MOI) for 48 h. β-actin was used as loading control and JJN3 treated with high doses of Bor as positive control (Cnt+). The histogram represents the protein bands intensity quantified using ImageJ software reported as arbitrary unit normalized by the loading control. **d** Western blot of Bcl-2 and Mcl-1 expression on JJN3 and OPM2 cells treated for 48 hours with or without BVDV (1 MOI). β-actin was used for loading control and RPMI-8226 cells line as positive control for both protein (Cnt+). The histograms represent the protein bands intensity quantified using ImageJ software reported as arbitrary unit normalized by the loading control. The *p* values were calculated by two-tailed Student’s *t* test. (*p < 0.05, **p < 0.01, ***p < 0.001) (CNT = control, untreated cells)

In order to further evaluate the cytotoxic effect of BVDV, we analyzed the expression of apoptotic markers. HMCLs treated with BVDV showed a significant increase of APO2.7 expression after 48 and 72 h of infection as compared to controls, as showed in Fig. 2a. The mean ± SD% of APO2.7 expression in HMCLs treated with BVDV vs. untreated cells was reported in Table 3. In Fig. 2b, we reported a representative experiment of APO2.7 staining on MM cells treated with BVDV for 24, 48, and 72 h. These results demonstrate that BVDV treatment increases the percentage of APO2.7^+^ cells over the time. Conversely, in non-MM cell lines, we did not find any differences in terms of APO2.7 expression between BVDV-treated cells and control conditions (Supplemental Figure S4). Furthermore, we found that the BVDV treatment of JJN3 and OPM2 cells for 48 h leads to the activation of caspase-3, and the downregulation of the anti-apoptotic proteins BCL-2 and MCL-1 (Fig. 2c, d). All these experiments showed that BVDV treatment reduced selectively the viability of MM cells by activating the apoptotic pathway.

**Table 3:** Statistical analysis of APO2.7 expression in HMCLs treated with BVDV

		Mean ± SD % of APO2.7 expression BVDV vs CNT	*p* value
JJN3	48 h	32.8 ± 6.1 vs. 13.02 ± 2.3	0.0009
	72 h	51.35 ± 6.8 vs. 17.4 ± 9.7	0.001
OPM2	48 h	12.6 ± 2.5 vs. 4.5 ± 0.6	0.0007
	72 h	39.7 ± 7.2 vs. 6.2 ± 1.8	0.0001
MM1.S	48 h	16.4 ± 1 vs. 8.4 ± 1.5	0.0001
	72 h	23.4 ± 1.7 vs. 10.4 ± 1.5	0.00003
NCI-H929	48 h	30.5 ± 3.3 vs. 10.5 ± 0.5	0.00002
	72 h	85.3 ± 3 vs. 10.2 ± 0.35	< 0.0001

Abbreviations: *BVDV* bovine viral diarrhea virus, *CNT* control (untreated cells), *SD* standard deviation

### Bortezomib pre-treatment increases the oncolytic effect of BVDV in HMCLs

**Fig 3.**
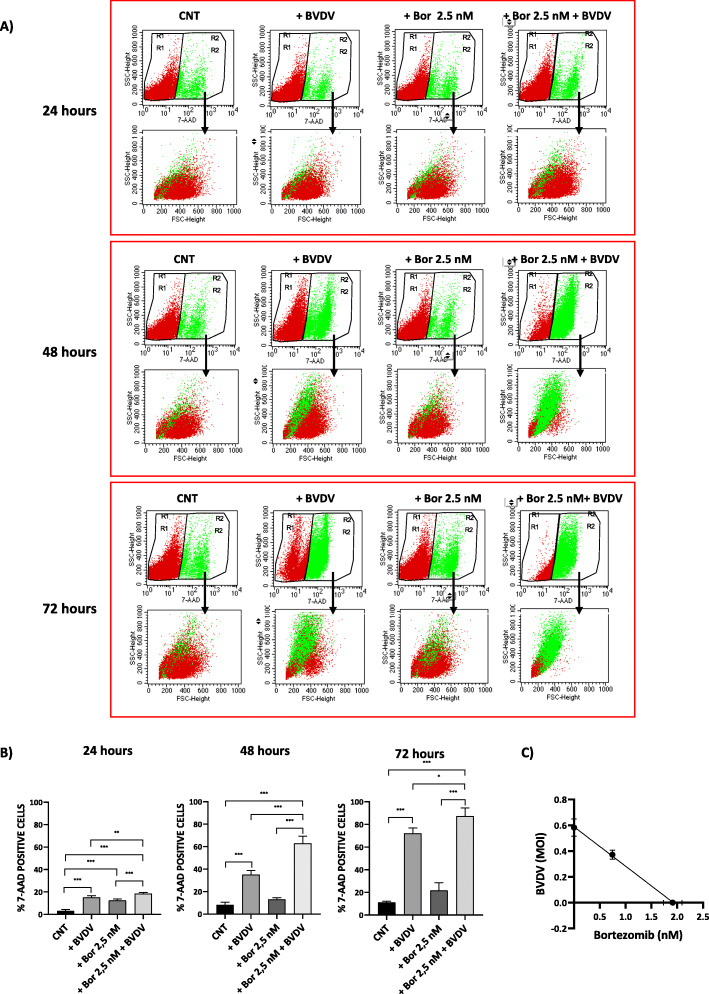
Pre-treatment with Bor increases the susceptibility of JJN3 to BVDV oncolytic activity. **a** Representative dot plots of flow cytometry analysis shown the percentages and morphology of viable (7-AAD^−^, red gate) and non-viable (7-ADD^+^, green gate) JJN3 cells after 24, 48, and 72 h of BVDV treatment (1 MOI), with or without 24 h of pre-treatment with Bor (2.5 nM). **b** The histograms represent the statistical analysis of four independent experiments of JJN3 cells pre-treated with Bor (2.5 nM) for 24 h and followed by BVDV treatment (1 MOI) for 24 (left panel), 48 (central panel), and 72 (right panel) h respectively. The *p* values were calculated by two-tailed Student’s *t* test. (*p < 0.05, **p < 0.01, ***p < 0.001) (CNT = untreated cells). **c** JJN3 cells were treated with increasing doses of Bor (from 0.125 to 8 nM), increasing doses of BVDV (from 0.0625 to 4 MOI), or the combination of the 2 drugs (2:1) or vehicle. After 48 h, cell viability was assessed, and the data were analyzed as % of the value obtained with the cells treated with vehicle. Combination index analysis was then performed using CompuSyn software. Isobologram for ED50 represents means ± SEM of 3 experiments with 5 determinations each

Because it has been reported that Bor increases the efficacy of several human oncolytic viruses in MM and other tumoral models [26–28], therefore, we tested MM cell death combining Bor and BVDV treatments. As reported in a representative sample in Fig. 3a, the Bor (2.5 nM) pre-treatment of JJN3 cells for 24 h enhances the cytotoxic in vitro effect of BVDV, increasing MM cell death over time. We observed a statistically significant decrease of cell viability after 24 and 48 h of BVDV treatment after Bor pre-treatment (mean ± SD% of 7-AAD^+^ dead cells: 24 h BVDV 15.22 ± 1.4 vs. Bor + BVDV 18.47 ± 1, *p* = 0.009; 48 h BVDV 35.06 ± 3.8 vs. Bor + BVDV 62.88 ± 6.4, *p* = 0.0003), reaching the highest mortality rates after 72 h (mean ± SD% of 7-AAD^+^ dead cells: 72 h BVDV 72.04 ± 4.8 vs. Bor + BVDV 87.25 ± 7.3, *p* = 0.013) (Fig. 3b).

Using Chou–Talalay analyses, we examined the drugs interaction between Bor and BVDV. JJN3 cells were pre-treated with various doses of Bor (0.125 nM–8 nM) for 24 h following the infection with different MOIs of BVDV for 48 h (0.0625–4 MOI). Viability data were then utilized to calculate the CI by the Compusyn program, in which CI < 1 indicates synergistic interaction and CI = 1 is additive. Our data showed that the combination of Bor and BVDV (at 2:1 ratio, respectively) synergistically killed MM cells. A synergistic effect was obtained for concentrations of Bor lower than 1.9 nM and of BVDV lower than 0.58 MOI, as shown for JJN3 in Fig. 3c. An additive effect was obtained for concentrations of Bor 1.9 nM and BVDV 0.58 MOI.

### Primary MM CD138^+^cells are susceptible to oncolytic activity of BVDV

**Fig. 4:**
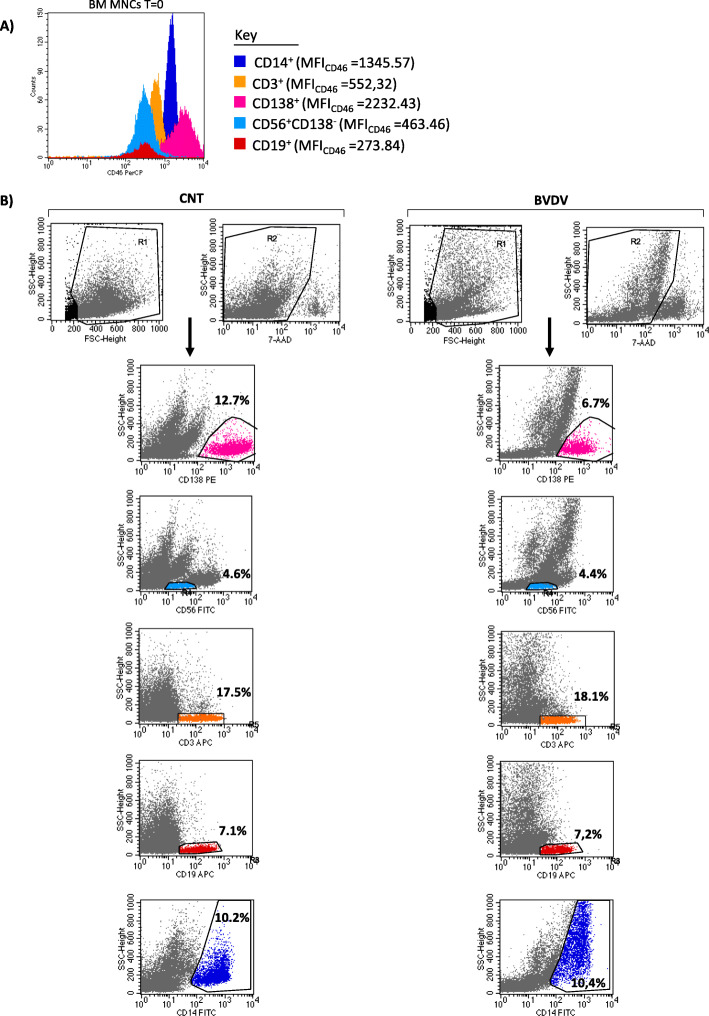
Expression levels of CD46 and ex vivo effect of BVDV on BM MNCs subpopulations **a** Flow cytometry histograms of one representative MM patient, showing the expression levels (MFI) of CD46 on monocytes (CD14^+^), T lymphocytes (CD3^+^), B lymphocytes (CD19^+^), NK cells (CD56^+^CD138^−^), and MM cells (CD138^+^). **b** Representative dot plots of flow cytometry analysis show the percentage of viable cells on BM subpopulations obtained from one MM patient after 72 h of BVDV (1 MOI) treatment compared to untreated control

We firstly analyzed the CD46 expression levels on the different BM subpopulations, as MM cells, monocytes, T, B, and natural killer (NK) lymphocytes. As expected, CD46 was expressed by all BM MNCs, but there was a marked heterogeneity in terms of the intensity of expression. In all samples, the flow cytometry analysis showed that the MFI of CD46 was higher on MM cells (CD138^+^) (median MFI_CD46_ value 1269.8, range 704.16–5149.88) in comparison with other subpopulations such as monocytes (CD14^+^), T lymphocytes (CD3^+^), NK (CD138^-^CD56^+^), and B lymphocytes (CD19^+^). Figure 4a reported the CD46 expression analysis on fresh BM MNCs from one representative MM patient (CD138^+^ MFI_CD46_ = 2232.43; CD14^+^ MFI_CD46_ = 1345.57; CD3^+^ MFI_CD46_ = 552.32; CD56^+^CD138^−^ MFI_CD46_ = 463.46; CD19^+^ MFI_CD46_ = 273.84). Subsequently, we investigated the BVDV ex vivo effect in 29 patients with active MM and from 2 patients with PCL after 72 h of treatment. As shown in a representative analysis of one MM patient (Fig. 4b), the BM MNCs treated with BVDV display a decrease of percentage of CD138^+^, while the other subpopulations remain unchanged.

**Fig. 5:**
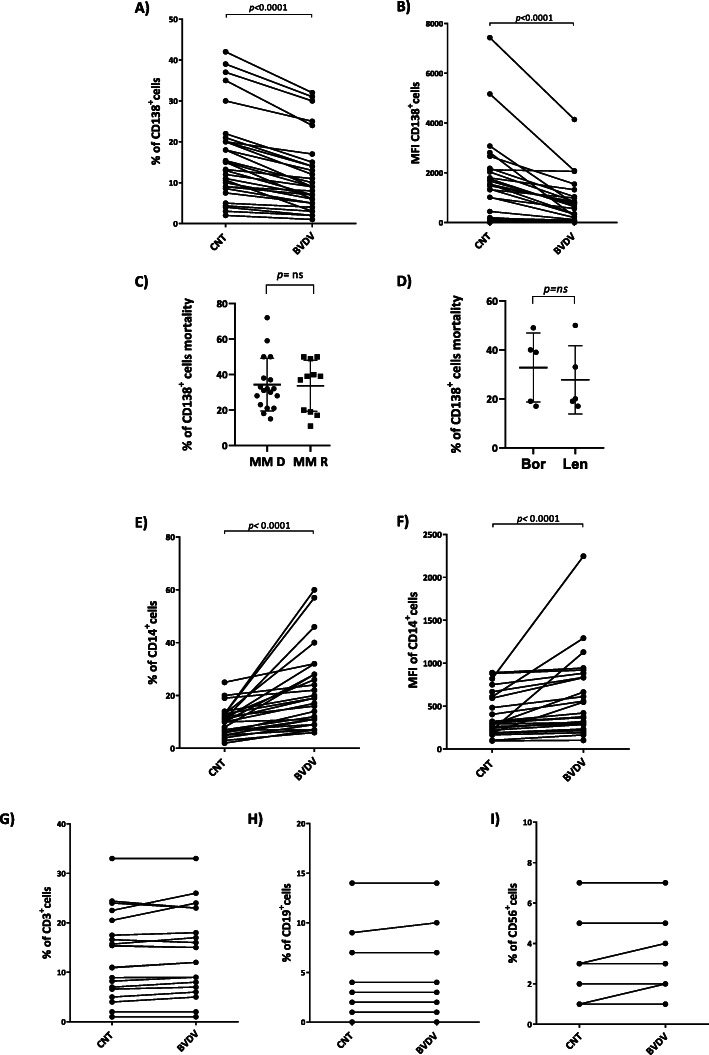
Ex vivo oncolytic activity of BVDV on CD138^+^ primary cells. The graphs represent the individual values of percentage (**a**) and MFI (**b**) of CD138^+^ cells obtained from BM MNCs of 31 patients treated with BVDV (1 MOI) for 72 h and in the untreated control. **c** The scatter plot displays the CD138^+^ cells mortality between BM MNCs from 18 patients with newly diagnosed MM (MM ND) and BM MNCs from 11 relapsed MM (MM R) patients; the analysis was performed as described in the “Methods” section. The *p* value was calculated by Mann-Whitney test (ns = not significant). **d** The scatter plot shown the CD138^+^ cells mortality between BM MNCs from 5 patients refractory to Bor treatment and BM MNCs from 5 Len-refractory patients; the analysis was performed as described in the “Methods” section. The *p* value was calculated by Mann-Whitney test (ns = not significant). The graphs show the percentage (**e**) and MFI (**f**) of CD14+ cells obtained from BM MNCs of 31 patients treated with BVDV (1 MOI) for 72 h and in the untreated control. The graphs represent the individual values of the percentage of CD3-positive cells (**g**) obtained from BM MNCs of 20 patients, the percentage of CD19-positive cells (**h**) obtained from BM MNCs of 16 patients and the percentage of CD56-positive cells (**i**) obtained from BM MNCs of 16 patients. All BM MNCs were treated with BVDV for 72 h or untreated as control condition. Paired sample are linked by a line. The *p* value was calculated by Wilcoxon’s test (CNT = control, untreated cells)

Analyzing our total cohort of BM MNCs from MM patients, we found a significant decrease of both the percentage of CD138^+^ cells (Fig. 5a) (*p* < 0.0001) and of the MFI_CD138_ (Fig. 5b) (*p* < 0.0001) after BVDV treatment compared to the control. Furthermore, considering patients with newly diagnosed MM and relapsed MM, we found that the BVDV-related mortality of CD138^+^ was not significantly different between two groups (Fig. 5c). Also between refractory patients to Bor or Lenalidomide (Len) treatment, we did not observed significantly differences in term of mortality cells of CD138^+^ cells (Fig. 5dc).

We also reported that the percentage of CD14^+^ increased after BVDV treatment (*p* < 0.0001) (Fig. 5e), also in terms of MFI_CD14_ (*p* < 0.0001) (Fig. 5f). Interestingly, we found that the percentage of CD3^+^, CD19^+^, and CD56^+^ cells, evaluated in a subset of our samples cohort after BVDV treatment, did not change (Fig. 5g–i). These results suggest that the BVDV oncolytic effect was limited to MM cells, potentially associated with a monocyte activation and did not affect lymphocyte populations.

### BVDV reduces tumor growth in vivo in NOD/SCID MM mouse model

**Fig. 6:**
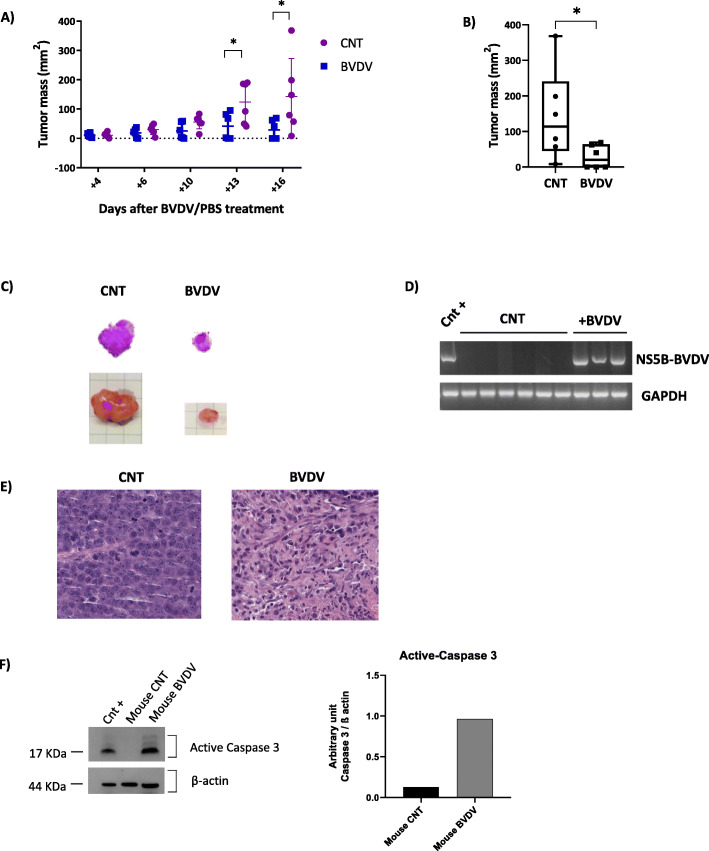
BVDV treatment inhibit tumoral growth in MM NOD/SCID mouse model. **a** Scatter plot represents the tumor mass (mm^2^) after 4, 6, 10, 13, and 16 days of intratumoral treatment with BVDV (blue dots) or PBS (CNT, violet dots) in mice with palpable plasmacytoma. Data are reported as individual values (plots) and the median range (bars). **b** Box plot graph reports the volumes of tumor mass collected after mice sacrifice in the untreated control condition and in the BVDV group. Values are reported as median volume and the range. *p* values were calculated by Mann-Whitney test. **c** Representative hematoxylin and eosin staining (top) and photographs of removed tumor masses (bottom) from one representative mouse of the control group and one of the BVDV-treated group (original magnification, × 1). **d** The picture shows the presence of BVDV in all the tumor treated of which was possible the collection after the end of the experiment (n°3), evaluated by Nested multiplex PCR. GAPDH was used as internal quality control. **e** Hematoxylin and eosin in staining of one tumor from the control group and one tumor from the mice treated with BVDV highlighting the tumor necrosis in the BVDV group (original magnification × 20). **f** Western blot of active-caspase 3 expression on plasmacytomas lysates obtained from one mouse treated with saline solution (mouse CNT) or one mouse treated with BVDV (mouse BVDV). β-actin was used as loading control and JJN3 treated with high doses of Bor as positive control (Cnt+). The histogram represents the protein bands intensity quantified using ImageJ software reported as arbitrary unit normalized by the loading control

Based on these in vitro results, we next evaluated the effect of BVDV treatment in an in vivo mouse model subcutaneously injected with JJN3 cells. Tumor volume measurements performed during treatment (at 4, 6, 10, 13, and 16 days after cells injection) showed a progressive reduction of tumor growth in mice treated with BVDV compared to controls (Fig. 6a). At the end of the experiment, we found that mice treated with BVDV showed a significant reduction of tumor masses as compared with untreated mice (*p* = 0.04) in terms of tumor volumes (Fig. 6b). A significant reduction of the tumors size was confirmed after plasmacytoma explant and hematoxylin-eosin staining, as shown for 2 representative mice in Fig. 6c. Interestingly, 3 mice out of 6 of the BVDV group showed a complete reduction and disappearance of tumor masses at the time of the mice sacrifice. The presence of BVDV has been assessed by multiplex PCR in all tumor masses treated with the bovine virus, where the plasmacytomas was still present at the end of the experiment as reported in Fig. 6d. Finally, the IHC analysis performed on tumor masses showed that the mice treated with BVDV presented necrotic tumor area as compared to control (Fig. 6e). Furthermore, we analyzed the protein levels of active-caspase 3 and β-actin by western blot on ex vivo plasmacytoma lysates from a representative mouse treated with BVDV or saline solution. Interestingly, we observed the activation of caspase-3 only in mouse treated with BVDV, showing that the reduced tumor mass is associated with apoptotic death of tumor cells (Fig. 6f).

## Discussion

Oncolytic virotherapy is an emerging therapeutic approach for MM as for other cancers [29, 30]. Most of the data published reported the use of human MV to kill MM cell [31]. More recently, other virus as reovirus, myxoma, and adenovirus were reported to have oncolytic activity in MM cells [12, 32, 33].

MV interacts with CD46 to enter into MM cells and to induce a cytopathic effect [15]. Actually, different MV constructs have been administrated to patients with MM in clinical trial with encouraging results [14, 34]. However, one of the main concerns regarding the use of MV as well as of other human virus is the presence of neutralizing anti-virus antibodies in cancer patients related to previous immunity. Indeed, the cases reported of an anti-MM effect of MV administration had undetectable circulating anti-MV antibodies. Because the vaccination anti-MV is a worldwide necessary procedure to protect from MV, infection alternative approach for oncolytic virus therapy should be found [35]. The Mayo Clinic approach considers different modalities to overcome the blocking activity of anti-MV antibodies including pre-therapy transiently depletion of anti-MV antibodies, the use of MV-infected cell carriers to deliver the virus evading anti-MV antibodies and the use of engineering oncolytic MV not recognized by anti-MV antibodies [35]. Despite these promising approaches, an alternative and innovative strategy for virotherapy could be the use of non-human virus. In this study, we tested this hypothesis, investigating the possible oncolytic activity in human MM cells of the BVDV a bovine pestivirus associated with mucosal disease in the caw. For the first time we demonstrated the oncolytic activity of this virus for tumor cells. We studied the BVDV, a non-pathogen virus for humans; it is known to bind CD46 to enter into cells, as showed for the MV [16]. We also tested in HMCLs the BoHV-4, a bovine virus known to have an oncolytic activity in tumor cells, but we did not find any significant cytopathic effect in MM cells. Interestingly, BoHV-4 is not able to bind CD46 as reported for the BVDV [16, 36], indicating that CD46 is critical for the oncolytic activity of BVDV.

CD46 is known to be expressed by all cell types, except erythrocytes [37–39]. Ong HT et al. showed that even though CD46 is ubiquitously expressed at low levels on all nucleated cells, it is expressed, quantitatively, at higher levels on MM cells compared to all other cellular populations in the BM [15], and it is considered a possible target either for virotherapy or for antibody-mediated immunotherapy [40, 41]. It was reported that MV infection induced cell death of several cancer cell lines other than MM cells [42], and that its efficacy was correlated to the level of CD46 expression by tumor cells [43]. In addition, it was recently reported that, in MM cells, CD46 expression was associated with p53 mutational status and that P53 mutated MM cells were highly sensitive to MV cytopathic effect [41]. Other authors reported a relationship between CD46 expression and the presence of 1q gain amplification in MM cells [40]. In our study, firstly, we confirmed the expression profile of CD46 on both HMCLs and primary MM cells and then we demonstrated the cytopathic activity of BVDV. This effect was independent by the presence of p53 mutational status of HMCLs and was attenuated by nutlin3a as reported by others [41]. Interestingly, we show that other cell lines, as acute leukemia and lymphoma, did not respond to the oncolytic effect of BVDV, despite their CD46 expression and BVDV ability to bind these cell lines. Overall, our results indicate that CD46 expression by tumor cells is necessary for the attachment of BVDV, but it is not sufficient to turn cells susceptible to infection and to achieve the oncolytic effect of BVDV, thus suggesting the involvement of other mechanisms. Literature data reported that Heparan sulfate family, including CD138 hallmark of MM, acts as a cellular receptor for BVDV binding to the host cells [36]. Our hypothesis is that other receptor/co-receptor, as CD138, could be involved in the mechanism of virus internalization into MM cells.

Several authors have reported that BVDV induces apoptosis in mammalian cells associated with the caspase-9 and caspase-8 activation that ultimately results in caspase-3 cleavage [20, 44, 45]. In line with literature data, our data show the cleavage of the effector caspases-3 in BVDV-treated MM cells. The activation of cellular caspase-3 on MM cells clearly correlated with the cytopathic BVDV-induced changes, suggesting a direct oncolytic effect of BVDV in MM cells mediated by apoptosis. In addition, beside caspase-3 activation, we found a significant downregulation of the BCL-2 and MCL-1 protein expression in BVDV-treated MM cells. As known BCL-2 proteins particularly MCL-1 are critically involved in the survival of MM cells [46–48]. However, appropriate studies will be necessary to clarify which transcriptional profile of MM cells as compared to other lymphoid cells is associated with the permissive role for BVDV in MM cells.

Data obtained on HMCLs were then confirmed in a large number of primary BM samples. Interestingly, we found that BVDV was able to induce a cytopathic effect independently by the type of primary sample tested either at the diagnosis or at the relapse. In all the BM samples tested, we found that CD138^+^ cells were only cell type susceptible to the oncolytic activity of BVDV, as demonstrated by the unchanged viability of CD14^+^, CD3^+^, CD19^+^, and CD56^+^ cells after BVDV treatment, despite their CD46 expression. These data interestingly suggest the lack of toxicity of the potential BVDV-based oncolytic virotherapy among BM cells. Along with the reduction of CD138^+^ viable cells, our results show that MM cells treated with BVDV displayed a significantly decrease in CD138 surface expression, thus suggesting its involvement in BVDV internalization. Moreover, these observations are in line with literature data showing a progressive loss of surface expression of CD138 on primary MM cells undergoing apoptosis [49]. Interestingly, we did not find any difference on BVDV effect between MM patients resistant or not to therapy and in addition between patients resistant to Bor or Len. These data suggested that the BVDV activity was independent to the presence of drug resistance in MM patients.

Based on the evidence of the oncolytic activity of BVDV in MM cells, following, we checked whether anti-MM drugs might improve the BVDV activity. Bor is a widely used proteasome inhibitor known to induce apoptosis through caspase-8 and caspase-9 signaling which further leads to caspase-3 activation in multiple myeloma cells [50]. Furthermore, several studies reported Bor ability to increase the oncolytic activity of different virus, as adenovirus and reovirus, in MM [51, 52]. In line with these observations, we showed that Bor pre-treatment significantly increase the oncolytic effect of BVDV with a synergistic effect due to the activation of the same apoptotic signaling, caspase-3-mediated. On the other hand, Len did not improve the oncolytic activity of the BVDV in MM cells (data not published).

Finally, to confirm the in vitro data, we tested the oncolytic activity of BVDV in an in vivo mouse model. We used a NOD/SCID mouse model to focus on the direct cytopathic effect of BVDV on MM cells using a subcutaneous route of administration of the virus. This preclinical model showed a significant in vivo anti-MM effect with a progressive reduction of tumor growth in mice treated with BVDV. In particular, we found that the reduced tumor mass is associated with caspase-3-mediated apoptotic death of tumor cells, confirming the in vitro and ex vivo data. Interestingly, we lack to find the presence of the virus in the vital organs as the heart and the lung indicating the high specificity of the BVDV for MM cells and the lack of toxicity (data not published).

## Conclusions

In conclusion, our results demonstrated for the first time an oncolytic activity of BVDV a bovine virus non-pathogen for human being showing that the BVDV oncolytic activity was specific for MM cells. Our data suggest that the use of BVDV is a possible alternative to human virus for an oncolytic approach in MM treatment. This study gives the rational to design clinical approach for the use of BVDV in patients with MM.

## Data Availability

All data generated or analyzed during this study are included in this published article [and its supplementary information files].

## Abbreviations

MM: Multiple myeloma
BVDV: Bovine viral diarrhea virus
PCs: Plasma cells
BM: Bone marrow
MV: Measles virus
HMCLs: Human myeloma cell lines
T-ALL: T-acute lymphoblastic leukemia
B-ALL: B-acute lymphoblastic leukemia
FBS: Fetal bovine serum
Bor: Bortezomib
PCL: Plasma cell leukemia
ND-MM: Newly diagnosed MM
R-MM: Relapsed MM
BoHV-4: Bovine herpesvirus-4 type 4
MNCs: Mononuclear cells
MFI: Median fluorescence intensity
